# Global change and human vulnerability to vector-borne diseases

**DOI:** 10.3389/fphys.2013.00158

**Published:** 2013-06-28

**Authors:** Rubén Bueno-Marí, Ricardo Jiménez-Peydró

**Affiliations:** Entomology and Pest Control Laboratory, Cavanilles Institute of Biodiversity and Evolutionary Biology, University of ValenciaValencia, Spain

This e-book presents a collection of research and review articles related to the spread, control and basic understanding of vector borne diseases all over the world. It is well known that a multidisciplinary point of view is necessary in order to develop a global vision of this emergent problem. Therefore, in order to promote this holistic approach to the knowledge of vector borne diseases, this e-book contains a total of 19 collaborations of entomologists, epidemiologists, virologists, parasitologists, bacteriologists, zoologists and veterinarians of Europe, Africa, Asia, and America. The title perfectly reflects some of the global factors that are behind the emergence and/or reemergence of vector borne diseases.

It is now well known that several climatic, environmental and sociodemographic changes that have occurred over the past years are some of the most important causes for the resurgence of many diseases worldwide. However, global change, defined as the impact of human activity on the fundamental mechanisms of biosphere functioning, includes not only climate change, but also habitat transformation, water cycle modification, biodiversity loss, synanthropic incursion of alien species into new territories, or the introduction of new chemicals in nature.

Although there is a large and varied group of vectors worldwide, in this e-book we have examined the two most important disease vectors in our opinion: mosquitoes and ticks. Studies about the presence and transmission rates of viruses like West Nile, assays about mosquito control with new and encouraging methods, studies related to the importance of vector control strategies, research results about the role of asymptomatic cases of anthroponosis like Dengue, and investigations about the impact of climate trends on diseases transmitted by ticks and mosquitoes, are some of the issues that can be found in this Research Topic.

As editors of this Research Topic, we would like to acknowledge sincerely all coauthors for their valuable and interesting contributions and we wish the readers of this e-book a productive and enjoyable reading of some of the most innovative work related to vector borne diseases.

